# Standardized brain tumor imaging protocols for clinical trials: current recommendations and tips for integration

**DOI:** 10.3389/fradi.2023.1267615

**Published:** 2023-12-13

**Authors:** Francesco Sanvito, Timothy J. Kaufmann, Timothy F. Cloughesy, Patrick Y. Wen, Benjamin M. Ellingson

**Affiliations:** ^1^ UCLA Brain Tumor Imaging Laboratory (BTIL), Center for Computer Vision and Imaging Biomarkers, University of California, Los Angeles, Los Angeles, CA, United States; ^2^ Department of Radiological Sciences, David Geffen School of Medicine, University of California, Los Angeles, Los Angeles, CA, United States; ^3^ Department of Radiology, Mayo Clinic, Rochester, MN, United States; ^4^ UCLA Neuro-Oncology Program, University of California, Los Angeles, Los Angeles, CA, United States; ^5^ Department of Neurology, David Geffen School of Medicine, University of California, Los Angeles, Los Angeles, CA, United States; ^6^ Center for Neuro-Oncology, Dana-Farber/Brigham and Women’s Cancer Center, Harvard Medical School, Boston, MA, United States; ^7^ Department of Bioengineering, Henry Samueli School of Engineering and Applied Science, University of California, Los Angeles, Los Angeles, CA, United States; ^8^ Department of Neurosurgery, David Geffen School of Medicine, University of California, Los Angeles, Los Angeles, CA, United States; ^9^ Department of Psychiatry and Biobehavioral Sciences, David Geffen School of Medicine, University of California, Los Angeles, Los Angeles, CA, United States

**Keywords:** brain tumors, neuro-oncology, clinical trials, brain tumor imaging protocols, BTIP, treatment response assessment, RANO criteria

## Abstract

Standardized MRI acquisition protocols are crucial for reducing the measurement and interpretation variability associated with response assessment in brain tumor clinical trials. The main challenge is that standardized protocols should ensure high image quality while maximizing the number of institutions meeting the acquisition requirements. In recent years, extensive effort has been made by consensus groups to propose different “ideal” and “minimum requirements” brain tumor imaging protocols (BTIPs) for gliomas, brain metastases (BM), and primary central nervous system lymphomas (PCSNL). In clinical practice, BTIPs for clinical trials can be easily integrated with additional MRI sequences that may be desired for clinical patient management at individual sites. In this review, we summarize the general concepts behind the choice and timing of sequences included in the current recommended BTIPs, we provide a comparative overview, and discuss tips and caveats to integrate additional clinical or research sequences while preserving the recommended BTIPs. Finally, we also reflect on potential future directions for brain tumor imaging in clinical trials.

## 1. General concepts

### 1.1. Role and aims of BTIPs in clinical trials

More than 80,000 patients are diagnosed with primary CNS tumors every year in the United States (incidence 24.7 per 100,000 population), with malignant forms accounting for 28.3% of the cases (1, 2). Among malignant primary tumors, gliomas have the highest incidence (4.26 per 100,000), while primary CNS lymphomas (PCNSL) are significantly rarer (0.46 per 100,000) (2). The incidence of metastatic CNS tumors is remarkably higher than primary tumors, with approximately 200,000 patients receiving a new diagnosis every year in the United States (3). Given the poor prognosis of these types of tumors, newer treatment options are constantly tested, including anti-angiogenic treatments, immunotherapy, and targeted therapy (3–7). Although the optimal endpoint of clinical trials for proving drug efficacy is an increase in overall survival (OS) in clinical trials, *radiologic* progression-free survival (PFS) and objective response rate (ORR) are considered valuable surrogate endpoints (8, 9). This concept applies to primary CNS tumors, and is even more relevant for patients with brain metastases (BM). In patients with BM, OS is frequently linked to systemic disease and the tested drugs may show heterogeneous efficacy on CNS localization compared to the systemic ones. For these two reasons, in BM patients the radiologic evaluation may be the most reliable measurement for drug efficacy in CNS.

One limitation of radiologic response assessment in clinical trials is the variability among images acquired in different institutions and on different scanners. Such variability arises from a number of factors including technique, acquisition parameters, two- vs. three-dimensional acquisition schemes, slice prescription and tilt/pitch differences, use of fat saturation, and timing of sequences with respect to the moment of gadolinium based contrast agent (GBCA) injection (10). In recent years, extensive effort has been made by consensus groups to propose standardized brain tumor imaging protocols (BTIPs), aiming to reduce such variability in measurement and interpretation (8, 11–13). The concept behind BTIPs is to ensure high image quality while maximizing the number of institutions meeting the acquisition requirements. Indeed, recommending ambitious guidelines that cannot be implemented in smaller institutions would dramatically reduce the number of centers eligible for clinical trials. As a partial solution for this compromise, BTIPs consensus papers feature both an “ideal” protocol and a “minimum” recommended protocol, with variations dependent on field strength. Additionally, proposed BTIPs comply to the will of limiting the protocols to 30 min, in order for them to be feasible in patients with low compliance on one hand, and compatible with the integration of additional clinically-required sequences on the other hand.

### 1.2. BTIPs serve RANO evaluation and beyond

The choice of BTIPs pulse sequences and the structure of the protocols are conceived to provide datasets whose evaluation can determine treatment response or failure as defined by the current recommended criteria. Treatment response in brain tumor clinical trials is assessed through Response Assessment in Neuro-Oncology (RANO) criteria. The first RANO criteria were originally proposed for high-grade gliomas (HGG), and are therefore often referred to as RANO-HGG (14). In the following decade, consensus groups proposed numerous variations and updates of RANO, including modified RANO (mRANO) for glioblastoma (15), specific criteria for lesions receiving immunotherapy (iRANO) (16), and for low-grade gliomas (LGG-RANO) (17). The latest effort in this regard is represented by the upcoming RANO 2.0, which aim to integrate previous considerations from different RANO guidelines and to address their reported limitations, to ultimately propose unified criteria (18). Of note, RANO 2.0 are conceived to be applicable to both LGG and HGG gliomas in adult patients, while brain metastases (BM) and pediatric patients may still be evaluated with dedicated criteria: RANO-BM (19) and RAPNO (20), respectively. Finally, separate response criteria have been proposed for PCNSL (21).

All variations of RANO criteria propose that, at every timepoint of the trial, each patient is assigned either progressive disease (PD), stable disease (SD), partial response (PR), or complete response (CR) compared to baseline or previous scans. The category assigned depends on the integrated evaluation of radiographic findings, clinical findings, and steroid dose. For the radiologic assessment, the most important sequence to evaluate is post-contrast T_1_-weighted (T_1_-post), since the enhancing tumor volume is the best radiologic surrogate of tumor burden in HGG (22–27), with some exceptions, and since BM tissue is exclusively enhancing. PCSNL radiologic evaluation, too, is based exclusively on enhancing lesions (21). Obviously, this does not apply to non-enhancing LGG, for which radiologic assessment is based on non-enhancing tumor volume (see LGG-RANO), evaluated on T_2_-weighted (T_2_) and T_2_-weighted fluid-attenuated inversion recovery (FLAIR). Notably, the evaluation of non-enhancing components in HGG is featured in RANO-HGG and iRANO, although no strict rules are mandated to perform such evaluation, while mRANO focus only on the enhancing tissue. The upcoming RANO 2.0 criteria propose to eschew the evaluation of non-enhancing components in tumors with contrast-enhancement, while preserving the evaluation of non-enhancing components in tumors that are largely-non-enhancing. The main role of pre-contrast T_1_-weighted (T_1_-pre) images is to exclude from the enhancing portion measurements any areas of spontaneous T_1_-hyperintensity (e.g., blood products, melanin). Interestingly, mRANO criteria propose a more thorough usage of T_1_-pre, by (optionally) evaluating the enhancement components based on T_1_-subtraction maps, which increase contrast-to-noise ratio of the enhancing tissue. T_1_-subtraction maps are obtained by normalizing T_1_-post and T_1_-pre signals, co-registering the two, and operating a voxel-wise subtraction (25). Finally, at this time none of the proposed RANO variations mandate an evaluation of diffusion MRI (dMRI) or perfusion MRI (pMRI), which are nonetheless included (dMRI) and encouraged (pMRI) in BTIPs, respectively.

While the main objective of the proposed BTIPs is to obtain high-quality images for response assessment in the present clinical trial, it must be emphasized that clinical trials represent an occasion to collect abundant longitudinal datasets from a patient population with set inclusion/exclusion criteria and with serial clinical evaluations. As such, images obtained during clinical trials are a valuable resource for subsequent retrospective radiologic studies on brain tumor patients, with the possibility of hypothesis testing with clinical and prognostic correlations. This should be taken into consideration when BTIPs are first implemented and potentially integrated with additional pulse sequences. Finally, BTIPs represent an occasion to reflect and reach consensus among panels of experts regarding the best strategies to obtain images to assess treatment efficacy. As such, concepts and guidelines emerging from BTIPs should also inspire and guide the choices of sequences and strategies for imaging protocols in the clinical routine.

## 2. Rationale behind BTIP sequences and technical considerations

### 2.1. T_1_-pre and T_1_-post images

As mentioned, the evaluation of the enhancing components with T_1_-weighted imaging is the most crucial step of the radiologic response assessment. The key concepts of T_1_-weighted imaging in BTIPs include matching parameters of T_1_-pre and T_1_-post, the timing, dosage, and type of contrast agent, the requirement for 3D imaging, and recommendations regarding the choice of gradient-echo (GRE) and/or spin-echo (SE) acquisitions (8, 11–13).

BTIPs recommend parameter-matched T_1_-pre and T_1_-post because acquiring both sequences with the same parameters and technique optimizes the comparative evaluation of enhancement with respect to inherent T_1_-pre signal, as well as the generation of T_1_-subtraction maps, if desired.

The timing of contrast injection is another relevant factor that can influence the evaluation. According to all BTIPs, T_1_-post images should be collected 4–8 min after contrast agent injection. This recommendation is based on previous evidence that the maximum contrast uptake takes place in this time window (28). Notably, for most lesions evaluated in this study the ratio between the enhancing tissue and normal gray matter reached its peak after 4–8 min and then plateaued for several minutes before slowly decreasing. Conversely, at 3.5 min 30% of lesions still hadn't reached their enhancement peak. Overall, this suggests that the highest lesion conspicuity is obtained by waiting at the very least 4 min from injection to T_1_-post acquisition, while waiting a little longer than 8 min may in theory not be as problematic. However, the 4–8 min time window should be respected also to maximize longitudinal reproducibility. This 4–8 min gap is typically filled with the acquisition of T_2_-weighted images. Additionally, the injection can be performed during the acquisition of dynamic susceptibility contrast (DSC) perfusion imaging. In this case, the time gap between injection and T_1_-post corresponds to the sum of the acquisition time of the post-bolus volumes of DSC and the acquisition time of the T_2_-weighted sequence.

BTIPs recommend to acquire T_1_-post after one *single* dose of GBCA of 0.1 mmol/kg. This corresponds to 1 ml/10 kg for contrast agents with molar concentration 1 mmol/ml, and 2 ml/10 kg for contrast agents with molar concentration 0.5 mmol/ml. This concept is important to keep in mind in case of integration with clinical or research protocols acquiring multiple boli of GBCA, for instance in case both DSC and DCE (dynamic contrast enhanced) imaging are performed, as discussed in the following paragraphs. On a related note, the commercially available gadolinium-based agents are characterized by remarkably different relaxivities, which impact the enhancement obtained for a given dose of contrast agent (29, 30). Therefore, the same type of GBCA should be used consistently at all follow-up scans for a given patient, to maximize the reliability of longitudinal comparisons, and should be reported on the DICOM header along with the dosage (8).

Moreover, 3D imaging is recommended. One of the reasons is that 3D imaging achieves thinner slices, which are known to allow a better evaluation of enhancement, including better detection of small lesions (31). 3D imaging also allows reformatting of the acquired imaging volume into other planes and to potentially adjust slice orientation. This is relevant because it has been shown that changes in head tilting can impact the treatment response evaluation (10). As an additional advantage, 3D imaging allows a better volumetric segmentation of the contrast-enhancing lesions. Improved segmentations have two advantages. First, they can be used to assess the radiologic response with volumetric thresholds as proposed by mRANO and RANO 2.0 (15, 18). Of note, the volumetric thresholds are applied to measurable disease, whose assessment is central for the response and progression criteria in RANO 2.0, together with the evaluation of the non-measurable disease, the clinical status, the steroid dosage, and the leptomeningeal involvement (18). Second, improved segmentations benefit further imaging analyses beyond clinical trials, for imaging studies focusing on tumor radiomics or advanced imaging.

Finally, BM and PCNSL BTIPs overall advocate that 3D TSE images should be preferred to 3D IR-GRE. This indication follows evidence supporting a better tumor-to-background contrast and lesion conspicuity using SE compared to GRE, when the slice thickness is comparable (31, 32). The historical reason for BTIPs including 3D IR-GRE (inversion recovery GRE, such as MPRAGE or IR-SPGR) is that on older scanners a thinner slice thickness is achievable with IR-GRE (8), and data showed that enhancement rate and contrast rate are higher for thin-sliced IR-GRE than for thick-sliced SE (31). When the slice thickness can be matched, though, and SE can be obtained with 1 mm voxels, the detectability of small lesions with SE is superior, as confirmed in a meta-analysis (33). This may be also ascribable to the high white matter signal in IR-GRE, which can mask small enhancing lesions for lack of tumor-to-background contrast. As an additional advantage, applying motion-sensitized driven-equilibrium preparation to TSE T_1_-post also allows black blood imaging (34), eliminating vascular enhancement that is typically seen in 3D IR-GRE and achieving a higher sensitivity in the identification of small superficial lesions of the cortex and leptomeningeal neoplastic involvement (35, 36). Finally, unlike IR-GRE, SE T_1_ allows for fat saturation, which is particularly helpful in case of metastastic involvement of the bony structures (12). Therefore, the recent BM and PCNSL BTIPs recommend employing pre- and post-contrast 3D TSE (turbo SE) in the “ideal” protocol, while leaving 3D IR-GRE in the “minimum” requirements together with an additional 2D SE T_1_ (12, 13), also considering that 3D TSE T_1_ superiority to 3D IR-GRE has been more thoroughly investigated at 3 T. As for gliomas, 3D IR-GRE is *currently* still the sequence of choice for T_1_-weighted imaging in clinical trials. In the original glioma BTIP initiative, 3D IR-GRE was preferred because it yields adequate image quality and is available on almost all MR systems as a result of the standardized ADNI initiative (37, 38), while 3D TSE is not available on all scanners and the various pulse sequences are not standardized across vendors. On a side note, adopting 3D TSE to improve lesion conspicuity and identify smaller lesions would be less relevant for gliomas *in clinical trials*, since, according to RANO 2.0, only *measurable* new contrast-enhancement (≥1 cm ×  ≥ 1 cm in-plane diameters) should be categorized as progressive disease. Overall, whether 3D TSE or 3D IR-GRE should be the T_1_-weighted sequence of choice for future glioma BTIPs is debated, as more institutions have been adopting 3D TSE in the clinical practice and pieces of evidence in its favor are being collected for gliomas (39). *As of today*, it is advisable to maintain 3D IR-GRE as the core T_1_-weighted sequence for gliomas to comply with glioma BTIP, with the option of acquiring additional post-contrast 3D TSE after, in case an institution has a preference toward it. However, this recommendation may change if a new agreement from a large panel of experts is reached.

### 2.2. T_2_-weighted images, T_2_-weighted FLAIR, and post-contrast T_2_-weighted FLAIR

T_2_-weighted TSE and T_2_-weighted FLAIR images are acquired to evaluate the non-enhancing components of tumors. As mentioned, the evaluation of non-enhancing tissue is central for LGG, marginal for HGG only according to RANO-HGG, iRANO, and RANO 2.0, and not acknowledged in mRANO, RANO-BM, and PCNSL response criteria. Overall, when the evaluation of non-enhancing sub-regions is required, response criteria do not dictate whether it should be performed using mainly T_2_ or FLAIR, and this choice is left to the preference of the reader, even though it is good practice to evaluate both.

Glioma BTIP proposes to optionally obtain T_2_ images through a dual-echo proton density and T_2_-weighted (PD/T_2_) TSE sequence (8). This approach allows to compute “effective T_2_”, a quantitative measurement of T_2_ relaxation with the potential of distinguishing vasogenic edema from non-enhancing tumor (40). While this quantitative metric is not currently used in response assessment, it has potential for the evaluation of non-enhancing tumor burden, and the acquisition of PD/T_2_ comes with no additional time penalty.

Both T_2_-weighted FLAIR and T_2_ images can be collected using 2D or 3D acquisition schemes. While 3D FLAIR has been encouraged since 2015 glioma BTIP (8), it only appeared as a required sequence in the recent PCNSL BTIP, in both the “ideal” and “minimum” protocols (13). The underlying reason is that, once again, BTIPs were structured to be inclusive of institutions with older scanners. However, 3D T_2_-weighted FLAIR images of good quality are progressively becoming more easily available on many scanners in use. As discussed for 3D T_1_, 3D T_2_-weighted FLAIR has the advantage of allowing tilting readjustment, reformatting in other planes, thinner slice thickness and better feasibility of lesion segmentation. For this reason, when images of good quality are obtainable, 3D FLAIR is highly recommended particularly for clinical trials involving LGG, where the assessment of the non-enhancing tumor is essential. Indeed, it has been recently demonstrated that 3D volumetric measurements obtained from lesion segmentation in LGG should be preferred for treatment response assessment, as they are have better inter-reader agreement and stable longitudinal measurements compared to 2D bidirectional diameters (41).

The use of fat saturation and the specific inversion time (TIs) prescribed are two aspects of FLAIR acquisition worth further discussion. The presence or absence of fat saturation can significantly impact the apparent extent and intensity of FLAIR signal alteration, constituting a potential confounding factor during interpretation of changes over time (10). Without the use of fat saturation, the bone marrow within the skull appears the brightest on T_2_-weighted FLAIR, while after fat saturation the bone marrow signal is nulled and T_2_ hyperintense areas are the brightest. Given the resulting images have a fixed dynamic range of signal intensities, the application of fat saturation can therefore dramatically change the contrast between non-enhancing tumor and surrounding brain tissue. Similar to other potential confounding factors (e.g., field strength, contrast agent type, scanning parameters), fat saturation is challenging to homogenize across institutions and the best practice is longitudinal consistency throughout all follow-up scans for a given patient. Currently, the PCNSL BTIP reports fat saturation on FLAIR as optional. As for TI, it has been shown that a lower TI (<2,400 ms at 3 T) enhances T_2_-FLAIR mismatch (T_2_FM) sign. T_2_FM is a radiogenomic sign with high specificity for IDH-mutant 1p19q-intact molecular status in gliomas (i.e., astrocytomas) when compared to IDH-mutant 1p19q-codeleted (i.e., oligodendrogliomas) and IDH-wild-type (i.e., glioblastomas), and arises from a partial T_2_ signal suppression on FLAIR images (42). The aforementioned study showed that a lower TI appears to increase the accuracy of T2FM to identify astrocytomas without causing an increase in false positive results (43). While a lower TI may improve the T_2_ signal suppression in some tumor regions for molecular profiling purposes, it also causes the corresponding tumor regions to appear isointense to normal tissue, with potential underestimation of non-enhancing tumor burden. Current BTIPs allow a certain flexibility when setting the TI (2,000–2,500 ms), with potential heterogeneity in T_2_ signal suppression in non-enhancing regions across different protocols. This once again advocates for evaluating both FLAIR and T_2_ during radiographic reads, if an assessment of the non-enhancing components is warranted, especially in protocols acquired with a higher FLAIR TI.

T_2_-weighted FLAIR images can also be acquired after the administration of contrast (FLAIR-post), which is preferred by some institutions and is featured in the PCNSL BTIP (13). FLAIR-post both display tissue with T_2_ hyperintensity and areas of contrast enhancement, since FLAIR images contain a mixture of both T_1_ and T_2_-weighting. In certain conditions such as low gadolinium concentration in the tissue, FLAIR-post has been reported to be more sensitive than T1-post in detecting subtle enhancement. FLAIR-post has potential applicability in several settings, including the detection of subtle intra-axial enhancement and the evaluation of lepto- and pachymeningeal neoplastic involvement (44). Such characteristics justify the inclusion of FLAIR-post in PCNSL BTIP, since lymphomas frequently disseminate to the meninges. Additionally, these characteristics support a potential application in gliomas and metastases to identify meningeal spread and for the detection of small intra-axial lesions (45–49), and some institutions prefer to acquire FLAIR-post in gliomas and BM, too. Acquiring FLAIR after contrast injection, as opposed to before, probably does not interfere with treatment response assessments. Indeed, the area of FLAIR intra-axial enhancement would lie within the T_2_-FLAIR hyperintense tissue, and therefore would not affect the measurement of the non-enhancing component in gliomas. As for meningeal spread, FLAIR-post may increase the sensitivity to meningeal involvement, which can be useful in both gliomas and BM. Overall, it is reasonable to consider FLAIR-post as an acceptable BTIL-compliant alternative in BM and gliomas—as long as protocol consistency is respected throughout timepoints. In the case FLAIR-post is preferred, the sequence order should be acquired as in PCNSL BTIP, with T_2_ before contrast and FLAIR after contrast. For efficiency, FLAIR-post should be acquired immediately after contrast agent injection per in PCNSL BTIP recommendations; however, data suggests contrast may be maximized through a delayed acquisition (20 min after contrast injection) (44), suggesting a potential role of collecting FLAIR-post *after* acquisition of conventional post-contrast T_1_-weighted images.

### 2.3. Diffusion and perfusion imaging

Diffusion-weighted imaging (DWI) and dynamic susceptibility contrast (DSC) perfusion imaging are common in both routine clinical and research studies in brain tumors (50–52).

DWI-derived apparent diffusion coefficient (ADC) images reflect microscopic water Brownian diffusion (53) and its values are considered a proxy for tumor cell density and tumor microstructure (52, 54). ADC has been proposed as a biomarker in neuro-oncology for molecular profiling (55–57), differential diagnosis (58), and treatment response assessment (54, 59). All current BTIPs propose 2D DWI acquisitions with at least 3 directions and at least 3 *b*-values (approximately 0, 500, and 1,000 s/mm^2^), per consensus recommendations by the International Society for Magnetic Resonance in Medicine (60).

While DWI is part of BTIPs and of the clinical neuroimaging protocols, ADC evaluation are currently *not* part of response assessment criteria. Part of the reason is that ADC interpretation is not trivial in the follow-up phase. Following treatment, and specifically radiation, changes in ADC values probably result from complex combined changes not only in cellularity, but also in extracellular matrix composition, in the presence of necrotic foci and edematous tissue, and possibly in vascular permeability. Additionally, ADC measurements suffer from susceptibility artifacts mainly induced by air-tissue interfaces and paramagnetic material (e.g., blood products) (61). Evidence from post-surgical case series (62) and from clinical trial images (63) suggest that artifacts and corrupted images may remarkably reduce the number of usable diffusion datasets, with a rate of images with unusable ADC values reported around 27.5% and 32%, respectively.

DSC perfusion is commonly employed in neuro-oncology to evaluate cerebral blood volume (CBV), and/or its derivates relative CBV and normalized relative CBV (rCBV and nrCBV, respectively), in order to quantify the degree of angiogenesis or tumor vascularity (64–66). Traditional measures of CBV has been shown to correlate with vascular density (67), thus providing a measure of relative tumor vascularity. Extensive literature has demonstrated the value of CBV for molecular profiling of gliomas (52, 55, 68), differential diagnosis (65, 69), treatment response assessment (70–72), and the discrimination between treatment effects and tumor recurrence (65, 73–75). From a technical standpoint, the estimation of CBV using DSC assumes the GBCA remains within the vasculature during acquisition (i.e., doesn't leak into the extravascular, extracellular space), and thus, the accuracy of CBV measurements is strongly affected by violations of this assumption (76). In particular, T_1_ leakage effects may result in CBV underestimation, therefore recent guidelines propose strategies to reduce CBV sensitivity to these effects (11). Current guidelines for DSC implementation in HGG include a combination of the following strategies: reducing the flip angle (FA) while using an appropriate field strength dependent echo time (TE), administering a preload bolus, and applying leakage correction during post-processing (11). FA and TE adjustments act on DSC sensitivity to T_1_ relaxation, since T_1_ sensitivity is mitigated by a low FA and/or high TE (76, 77). The administration of a preload bolus is aimed to partially saturate the baseline T_1_ contribution to the signal, therefore mitigating T_1_ leakage effects (11). Finally, post-hoc leakage correction using mathematical modeling to account for contrast leakage should be performed to further improve the accuracy of the measurements (11, 78). The current consensus guidelines for DSC on HGG recommend GRE echo planar imaging (GRE-EPI), either with a full preload GBCA dose (1 + 1 dosing) and FA 60° (“intermediate” FA) or with no preload (0 + 1 dosing) and FA 30° (“low” FA), with TE 30 ms (at 3 T) or 45 ms (at 1.5 T), and uni- or bidirectional leakage correction (11). BM and PCNSL BTIPs adopted DSC HGG guidelines and comply with the DSC consensus by proposing the alternative with low FA and no preload, if the 0 + 1 dosing is desired (12, 13). It is worth mentioning that only some of the previously proposed preload schemes are BTIP-compliant. The 0 + 1 (no preload), 1 + 1 (full dose preload) and ½ + ½ (half dose preload and half dose injection) schemes are all BTIP-compliant because they allow the acquisition of T_1_-post after a single dose of GBCA. Other schemes, such as ½ + 1 dosing, are not acceptable because T_1_-post would be acquired either after half dose or after one and a half.

In addition to CBV, DSC perfusion can potentially provide additional information about the tumor microenvironment. The comparison of post-bolus to pre-bolus DSC signal intensity, as measured with the percentage of signal recovery (PSR), is thought to be influenced by tissue microstructure, and therefore useful for differential diagnosis (65, 79). Post-bolus signal intensity is influenced by the balance between T_2_* and T_1_ leakage effects, which has been suggested to reflect tissue cytoarchitecture (77, 80, 81). PSR utility has been demonstrated in the diagnostic phase rather than in treatment response monitoring so far, thus its evaluation may not be directly relevant for clinical trials at the moment. However, it is worth noticing that a recent study supported the validity of PSR even when derived from BTIP-compliant DSC protocols that are optimized for CBV computation and therefore bear a weaker T_1_-weighting (82). This is relevant because it supports the adoption of BTIP-compliant DSC protocols for both CBV and PSR, both in the diagnostic and follow-up phases.

Although DSC is part of the clinical work-up of brain tumor and included in BTIPs, CBV assessment is *not* integrated in response criteria. During the monitoring of treatment response, CBV is potentially useful in cases of pseudoprogression (74, 83, 84), where tumor size assessment is not a reliable surrogate of tumor burden, since in such cases there is discrepancy between the apparent size changes and the actual response to the treatment. Another potential application of CBV in clinical trials is the demonstration of angiogenesis reduction following antiangiogenetic treatment (85, 86), even though such reduction does not appear to predict an extended OS (85). However, no reliable quantitative CBV cutoffs have been validated to identify disease progression. This is due to most studies being small and single center, inhomogeneity in DSC protocols, and a modest repeatability and reproducibility of CBV measurements (87). Overall, more efforts to achieve reliable and reproducible perfusion assessments are warranted in order to test CBV evaluations in multicenter trials.

## 3. Overview of current BTIPs

Table 1 provides a comparative overview of “ideal” recommended BTIPs at 3 T for gliomas (8), BM (12), and PCNSL (13). Table 2 illustrates the corresponding “minimum” BTIPs at 1.5 T. While the tables compare the protocols at the extreme of the spectrum (ideal at 3 T and minimum at 1.5 T), the cited BTIPs also propose “minimum” protocols at 3 T (only BM and PCNSL BTIPs) or an “ideal” protocol at 1.5 T (only glioma BTIP) (not reported in the tables). In both tables, DSC recommendations for gliomas have been integrated from the separate consensus paper for DSC in HGG (11).

**Table 1:** Comparison of ideal BTIPs at 3 T for gliomas, PCNSL, and BM.

Gliomas	T_1_-pre	FLAIR^a^	DWI^b^	DSC^c^	T_2_	T_1_-post^d^
Sequence	***IR-GRE***	TSE	EPI	GRE-EPI	TSE	***IR-GRE***
Plane	Sag/ax	Ax	Ax	Ax	Ax	Ax/sag
Mode	3D	2D	2D	2D	2D	3D
TR [ms]	2,100 Si/Hi; 5–15 GE/Phi/To	>6,000	>5,000	1,000–1,500	>2,500	2,100 Si/Hi; 5–15 GE/Phi/To
TE [ms]	Min	100–140	Min	25–30 for 30° FA 20–35 for 60° FA	80–120	Min
TI [ms]	1,100 Si/Hi; 400–450 GE/Phi/To	2,500				1,100 Si/Hi; 400–450 GE/Phi/To
FA	10°–15°	90°/≥160°	90°/180°	60°/30° w/ preload 30° w/o preload	90°/≥160°	10°–15°
Frequency	256	≥256	128	128 or ≥96	≥256	256
Phase	256	≥256	128	128 or ≥96	≥256	256
NEX	≥1	≥1	≥1	1	≥1	≥1
FOV	256 mm	240 mm	240 mm	220–240 mm	240 mm	256 mm
Slice thickness	1 mm	3 mm	3 mm	3–5 mm	3 mm	1 mm
Gap/spacing	0	0	0	0–1 mm	0	0
Options			*b *= 0, 500, 1,000 s/mm^2^ ≥ 3 directions	30–50 tp before bolus; ≥120 total tp		
Parallel imaging	Up to 2x	Up to 2x	Up to 2x	Up to 2x	Up to 2x	Up to 2x
Notes		Pre-CA		Adjust FA if preload	Post-CA 1st	Post-CA 2nd
BM	T_1_-pre	FLAIR^a^	DWI^b^	DSC	T_2_	T_1_-post
Sequence	***TSE***	TSE	SS-EPI	GRE-EPI	TSE	***TSE***
Plane	sag/ax	Ax	Ax	Ax	Ax	sag/ax
Mode	3D	2D	2D	2D	2D	3D
TR	550–750	>5,000	>5,000	1,000–1,500	>2,500	550–750
TE	Min	100–140	Min	25–35	80–120	Min
TI		2,500 3T				
FA	Default	90°/≥160°	90°/180°	30°	90°/≥160°	Default
Frequency	256	≥256	128	≥96	≥256	256
Phase	256	≥256	128	≥96	≥256	256
NEX	≥1	≥1	≥1	1	≥1	≥1
FOV	256 mm	240 mm	240 mm	240 mm	240 mm	256 mm
Slice thickness	1 mm	3 mm	3 mm	3–5 mm	3 mm	1 mm
Gap/spacing	0	0	0	0–1 mm	0	0
Options			*b *= 0, 500, 1,000 s/mm^2^ ≥ 3 directions	30–60 tp before bolus; >120 total tp		
Parallel imaging	Up to 3x	Up to 2x	Up to 2x	Up to 2x	Up to 2x	Up to 3x
Notes		Pre-contrast		***No preload***	Post-CA 1st	Post-CA 2nd
PCNSL	T_1_-pre	FLAIR-post^e^	DWI^b^^,^^e^	DSC	T_2_^e^	T_1_-post
Sequence	***TSE***	TSE	SS-EPI	GRE-EPI	TSE	***TSE***
Plane	Any	Any	Ax	Ax	Ax	Any
Mode	3D	***3D***	2D	2D	***3D***	3D
TR	550–750	>6,000	>5,000	1,000–1,500	>2,500	550–750
TE	Min	90–140	Min	25–35	80–120	Min
TI		2,000–2,500				
FA	Default	90°/≥160°	90°/180°	30°	90°/≥160°	Default
Frequency	256	≥256	128	≥96	≥256	256
Phase	256	≥256	128	≥96	≥256	256
NEX	≥1	≥1	≥1	1	≥1	≥1
FOV	256 mm	240 mm	240 mm	240 mm	240 mm	256 mm
Slice thickness	1 mm	***1 mm***	3 mm	3–5 mm	***1 mm***	1 mm
Gap/spacing	0	0	0	0–1 mm	0	0
Options	***Fat sat optional***	***Fat sat optional***	*b *= 0, 500, 1,000 s/mm^2^ ≥ 3 directions	30–60 tp before bolus; >120 total tp		***Fat sat optional***
Parallel imaging	Up to 2x	Up to 2x	Up to 2x	Up to 2x	Up to 2x	Up to 2x
Notes		***Post-CA 1st***	As first sequence	***No preload***	***Pre-CA***	Post-CA 2nd

The most relevant differences between protocols are highlighted with bold. CA, contrast agent; Si, siemens; Hi, hitachi; GE, general electric; Phi, philips; To, toshiba; tp, timepoints.

^a^ Acquiring FLAIR post-contrast, if preferred, by inverting the order of T_2_ and FLAIR as in the PCNSL BTIP could be a reasonable variation to be considered BTIP-compliant overall (see text for discussion).

^b^ DTI can be considered a BTIP-compliant alternative to DWI (see text for discussion).

^c^ DSC recommendations for gliomas are integrated from the separate consensus paper and DSC was considered optional in the original HGG BTIP.

^d^ In gliomas, if 3D TSE is preferred, it is *currently* advisable to acquire both 3D IR-GRE (first) and 3D TSE (second) to maintain glioma BTIP compliance (see text for discussion).

^e^ For an easier comparison across protocols, DWI, FLAIR and T_2_ are *not* displayed in chronological order in this PCNSL BTIP overview, as T_2_ is acquired pre-contrast, FLAIR post-contrast, and DWI is proposed as first sequence of the protocol.

**Table 2:** Comparison of minimum BTIPs at 1.5 T for gliomas, PCNSL, and BM.

Gliomas	T_1_-pre	FLAIR^a^	DWI^b^	DSC (optional)^c^	T_2_		T_1_-post^d^
Sequence	***IR-GRE***	TSE	SS-EPI	GRE-EPI	TSE		***IR-GRE***
Plane	Sag/ax	Ax	Ax	Ax	Ax		Sag/ax
Mode	3D	2D	2D	2D	2D		3D
TR [ms]	2,100 Si/Hi; 5–15 GE/Phi/To	>6,000	>5,000	1,000–1,500	>2,500		2,100 Si/Hi; 5–15 GE/Phi/To
TE [ms]	Min	100–140	Min	40–50	80–120		Min
TI [ms]	1,100 Si/Hi; 400–450 GE/Phi/To	2,000					1,100 Si/Hi; 400–450 GE/Phi/To
FA	10°–15°	90°/≥160°	90°/180°	60°/30° w/ preload 30° w/o preload	90°/≥160°		10°–15°
Frequency	≥172	≥256	≥128	≥96	≥256		≥172
Phase	≥172	≥256	≥128	≥96	≥256		≥172
NEX	≥1	≥1	≥1	1	≥1		≥1
FOV	256 mm	240 mm	240 mm	220–240 mm	240 mm		256 mm
Slice thickness	≤1.5 mm	≤4 mm	≤4 mm	4–5 mm	≤4 mm		≤1.5 mm
Gap/spacing	0	0	0	0–1 mm	0		0
Options			*b *= 0, 500, 1,000 s/mm^2^ ≥ 3 directions	30–50 tp before bolus; ≥120 total tp			
Parallel imaging	Up to 2x	Up to 2x	Up to 2x	Up to 2x	Up to 2x		Up to 2x
Notes		Pre-CA		Adjust FA if preload	Post-CA 1st		Post-CA 2nd
BM	T_1_-pre	FLAIR^a^	DWI^b^	DSC (optional)^c^	T_2_	T_1_-post add^e^	T_1_-post
Sequence	***IR-GRE***	TSE	SS-EPI	GRE-EPI	TSE	***TSE/SE***	***IR-GRE***
Plane	Sag/ax	Ax	Ax	Ax	Ax	Ax/Cor	Sag/ax
Mode	3D	2D	2D	2D	2D	***2D***	3D
TR	2,100 Si/Hi; 5–15 GE/Phi/To	>6,000	>5,000	1,000–1,500	***>3,500***	400–600	2,100 Si/Hi; 5–15 GE/Phi/To
TE	Min	100–140	Min	40–50	80–120	Min	Min
TI	1,100 Si/Hi; 400–450 GE/Phi/To	2,000					1,100 Si/Hi; 400–450 GE/Phi/To
FA	10°–15°	90°/≥160°	90°/180°	60°/30° w/ preload 30° w/o preload	90°/≥160°	90°/≥160°	10°–15°
Frequency	≥172	≥256	128	≥96	≥256	≥256	≥172
Phase	≥172	≥256	128	≥96	≥256	≥256	≥172
NEX	≥1	≥1	≥1	1	≥1	≥1	≥1
FOV	256 mm	240 mm	240 mm	220–240 mm	240 mm	240 mm	256 mm
Slice thickness	≤1.5 mm	≤4 mm	≤4 mm	4–5 mm	≤4 mm	≤4 mm	≤1.5 mm
Gap/spacing	0	0	0	0–1 mm	0	0	0
Options			*b *= 0, 500, 1,000 s/mm^2^ ≥ 3 directions	30–50 tp before bolus; ≥120 total tp		***Fat sat encouraged***	
Parallel imaging	Up to 2x	Up to 2x	Up to 2x	Up to 2x	Up to 2x	Up to 2x	Up to 2x
Notes		Pre-CA		Adjust FA if preload	Post-CA 1st	Post-CA 2nd	Post-CA 3rd
PCNSL	T_1_-pre	FLAIR-post^f^	DWI^b^^,^^f^	DSC	T_2_^f^	T_1_-post add^e^	T_1_-post
Sequence	***IR-GRE***	TSE	SS-EPI	GRE-EPI	TSE	***TSE/SE***	***IR-GRE***
Plane	Sag/ax	Any	Ax	Ax	Any	Ax/Cor	Sag/ax
Mode	3D	***3D*** ^g^	2D	2D	***3D*** ^h^	***2D***	3D
TR	2,100 Si/Hi; 5–15 GE/Phi/To	>6,000	>5,000	1,000–1,500	>2,500	400–600	2,100 Si/Hi; 5–15 GE/Phi/To
TE	Min	90–140	Min	45	80–120	Min	Min
TI	1,100 Si/Hi; 400–450 GE/Phi/To	2,000–2,500					1,100 Si/Hi; 400–450 GE/Phi/To
FA	10°–15°	90°/≥160°	90°/180°	30–35°	90°/≥160°	90°/≥160°	10°–15°
Frequency	172	≥256	128	≥96	≥256	≥256	172
Phase	172	≥256	128	≥96	≥256	≥256	172
NEX	≥1	≥1	≥1	1	≥1	≥1	≥1
FOV	256 mm	240 mm	240 mm	240 mm	240 mm	240 mm	256 mm
Slice thickness	≤1.5 mm	≤***1.5 mm***	≤4 mm	3–5 mm	≤***1.5 mm***	≤4 mm	≤1.5 mm
Gap/spacing	0	0	0	0–1 mm	0	0	0
Options		***Fat sat optional***	*b *= 0, 500, 1,000 s/mm^2^ ≥ 3 directions	30–60 tp before bolus; >120 total tp		***Fat sat optional***	*** ***
Parallel imaging	Up to 2x	Up to 2x	Up to 2x	Up to 2x	Up to 2x	Up to 2x	Up to 2x
Notes		***Post-CA 1st***	As first sequence	***No preload***	***Pre-CA***	Post-CA 2nd	Post-CA 3rd

The most relevant differences between protocols are highlighted with bold. CA, contrast agent; Si, siemens; Hi, hitachi; GE, general electric; Phi, Philips; To, toshiba; tp, timepoints; add, additional.

^a^ Acquiring FLAIR post-contrast, if preferred, by inverting the order of T_2_ and FLAIR as in the PCNSL BTIP could be a reasonable variation to be considered BTIP-compliant overall (see text for discussion).

^b^ DTI can be considered a BTIP-compliant alternative to DWI (see text for discussion).

^c^ DSC is optional for gliomas and BM, and not part of the minimum requirements in the original HGG BTIP and in the BM BTIP. Even though the separate consensus paper for DSC was meant for HGG, it is reasonable to integrate the same DSC guidelines for both HGG and BM, as in this table.

^d^ In gliomas, if TSE/SE is desired, it is *currently* advisable to acquire both 3D IR-GRE (first) and TSE/SE (second) to maintain glioma BTIP compliance (see text for discussion).

^e^ In BM and PCNSL BTIPs, the recommendation is to acquire the additional T_1_-post SE 2D *before* the parameter-matched IR-GRE 3D T_1_-post.

^f^ For an easier comparison across protocols, DWI, FLAIR and T_2_ are *not* displayed in chronological order in this PCNSL BTIP overview, as T_2_ is acquired pre-contrast, FLAIR post-contrast, and DWI is proposed as first sequence of the protocol.

^g^ 2D FLAIR is accepted as an alternative in PCNSL BTIP. If adopted, it should be set as in the BM BTIP.

^h^ If 3D T_2_ is not available for PCNSL BTIP, 2D T_2_ should be acquired with minimal slice thickness.

As previously mentioned, 3D IR-GRE T_1_ is featured in the glioma BTIP, while 3D TSE T_1_ sequences are recommended as ideal in PCNSL and BM BTIPs. In case 3D TSE T_1_ are not available, it is suggested to use 3D IR-GRE T_1_-pre and acquire an additional 2D SE T_1_-post before 3D IR-GRE T_1_-post. When both a 3D IR-GRE and a 2D SE T_1_-post are acquired according to the minimum requirement protocols, it is advisable to use 2D SE T_1_-post to detect *new* non-measurable intra-axial lesions and osseous involvement, thanks to the superior conspicuity of small lesions on SE and to the possibility of fat saturation. On the other hand, if SE T_1_-post is acquired 2D, it is advisable to base lesion *measurements* on 3D IR-GRE, as it is parameter-matched with T1-pre, allowing for a better pre-to-post comparison and to exclude from the measurements the spontaneous T_1_-hyperintensity (e.g., due to hemorrhage or melanin), whether T_1_-subtraction maps are employed or not.

Other differences include 3D imaging for FLAIR and T_2_ in the most recent PCNSL BTIP, although 3D FLAIR was already strongly recommended in the glioma BTIP. Additionally, 3D FLAIR is acquired after contrast (FLAIR-post) according to the PCNSL BTIP, which eliminates the need for T_2_ being acquired after contrast injection.

Overall, the ideal protocols and a higher magnetic field (3 T) should be preferred, if possible. However, large-scale multicenter clinical trials often involve smaller academic and non-academic community-based hospitals, where only the “minimum” protocols may be feasible. In such cases, it is advisable, for the sake of consistency and standardization, that all the centers involved in the same trial comply with MRI protocols applicable to all the trial locations. As a result, most later stage clinical trials may choose to adopt the “minimum” protocols, whereas the “ideal” protocols designed for high-performance 3 T scanners may be more applicable in smaller, early phase studies at specific academic institutions.

**Figure 1:**
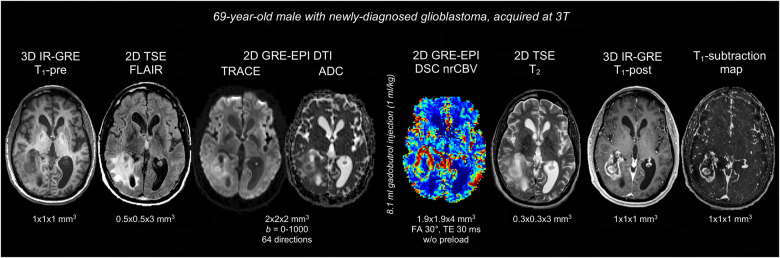
Representative case of BTIP-compliant glioma MRI exam. The images are shown in chronological order, and are displayed in their native acquisition space and voxel size. T_1_ images are acquired with matching technique (3D IR-GRE), parameters and voxel-size pre- and post-contrast, allowing for optimal T_1_-subtraction maps. FLAIR and T_2_ images are acquired with 2D TSE sequences. Diffusion imaging is obtained through a DTI acquisition (*b *= 0–1,000; 64 directions), as an alternative to axial DWI at 3 directions. DSC perfusion imaging is acquired without preload with a 30° flip-angle, in compliance with DSC guidelines.

**Figure 2:**
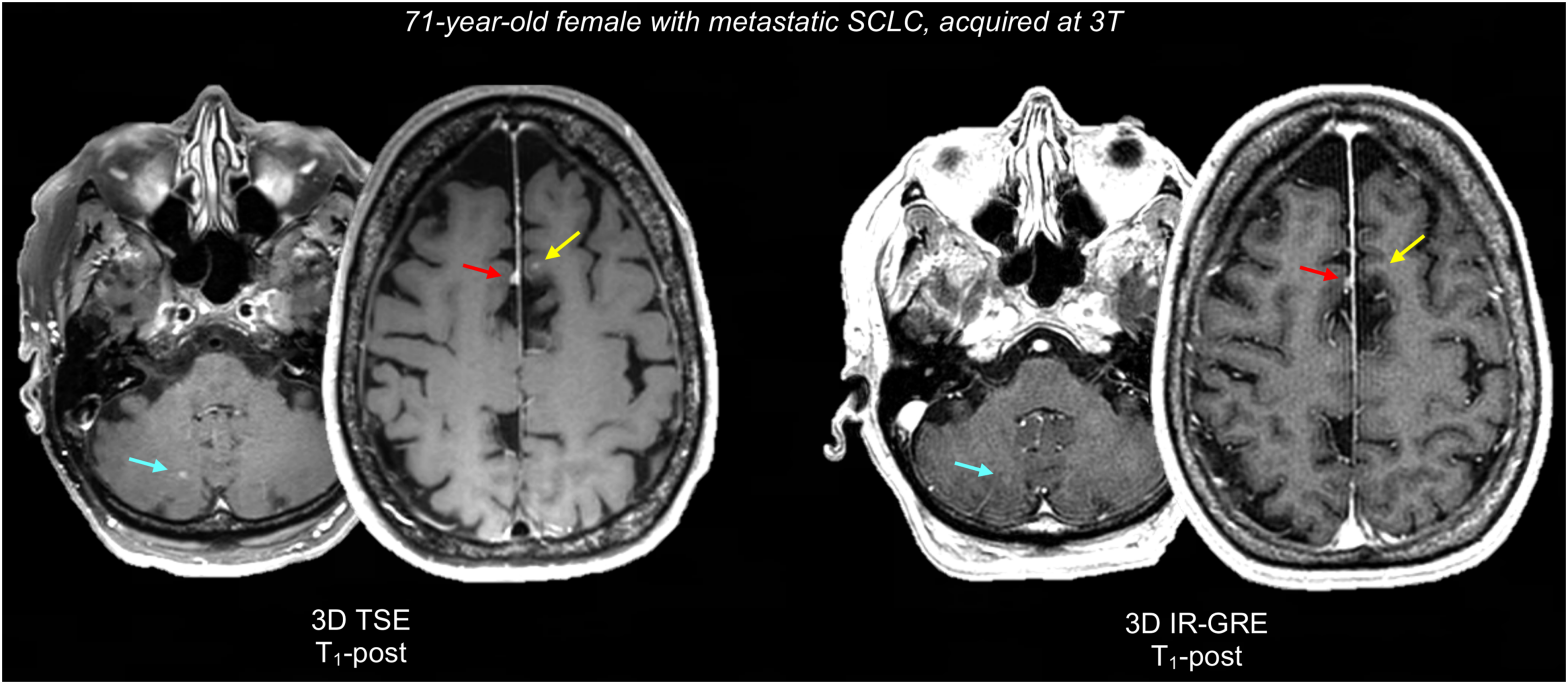
Representative case comparing lesion conspicuity between 3D TSE and 3D IR-GRE T_1_-post in a BM patient. 3D IR-GRE was acquired for SRS planning, one day after 3D TSE. 3D TSE shows a juxtacortical BM in the left superior frontal gyrus (2 mm, yellow arrow), that is not appreciable on 3D IR-GRE. Similarly, a right paravermian cerebellar BM (3 mm, teal arrow) is easily identifiable on 3D TSE, while it barely appreciable on 3D IR-GRE. Finally, both sequences show an incidental parafalcine meningioma (red arrow), which is also more conspicuous on 3D TSE, probably due to the reduced number of confounding bright cortical vessels. SCLC, small cell lung carcinoma.

Figure 1 displays a representative BTIP-compliant MRI exam for a glioma case. Figure 2 shows demonstrative images of improved lesion conspicuity on TSE T_1_-post compared to IR-GRE T_1_-post in a BM patient.

## 4. Integration of additional sequences

BTIPs are typically acquired in the same MRI session as imaging needed for the clinical management of the patients and potentially also together with research sequences, especially in academic institutions. Therefore, it is important to be aware of *caveats* to be observed when integrating BTIPs and other clinical and research pulse sequences.

A crucial aspect to consider is the timing and dosage of GBCA administration with respects to additional sequences and BTIP sequences. Some additional sequences must be obtained before GBCA (e.g., functional MRI, fMRI), some others can be obtained either before or after (e.g., sodium imaging), and other ones require an additional GBCA administration (e.g., DCE). As for BTIP sequences, protocols integrating clinical or research acquisitions should be BTIP-compliant, meaning they should observe that T_1_-post images must be acquired around 4–8 min after contrast injection and after exactly one *single* dose of GBCA (0.1 mmol/kg).

**Figure 3:**
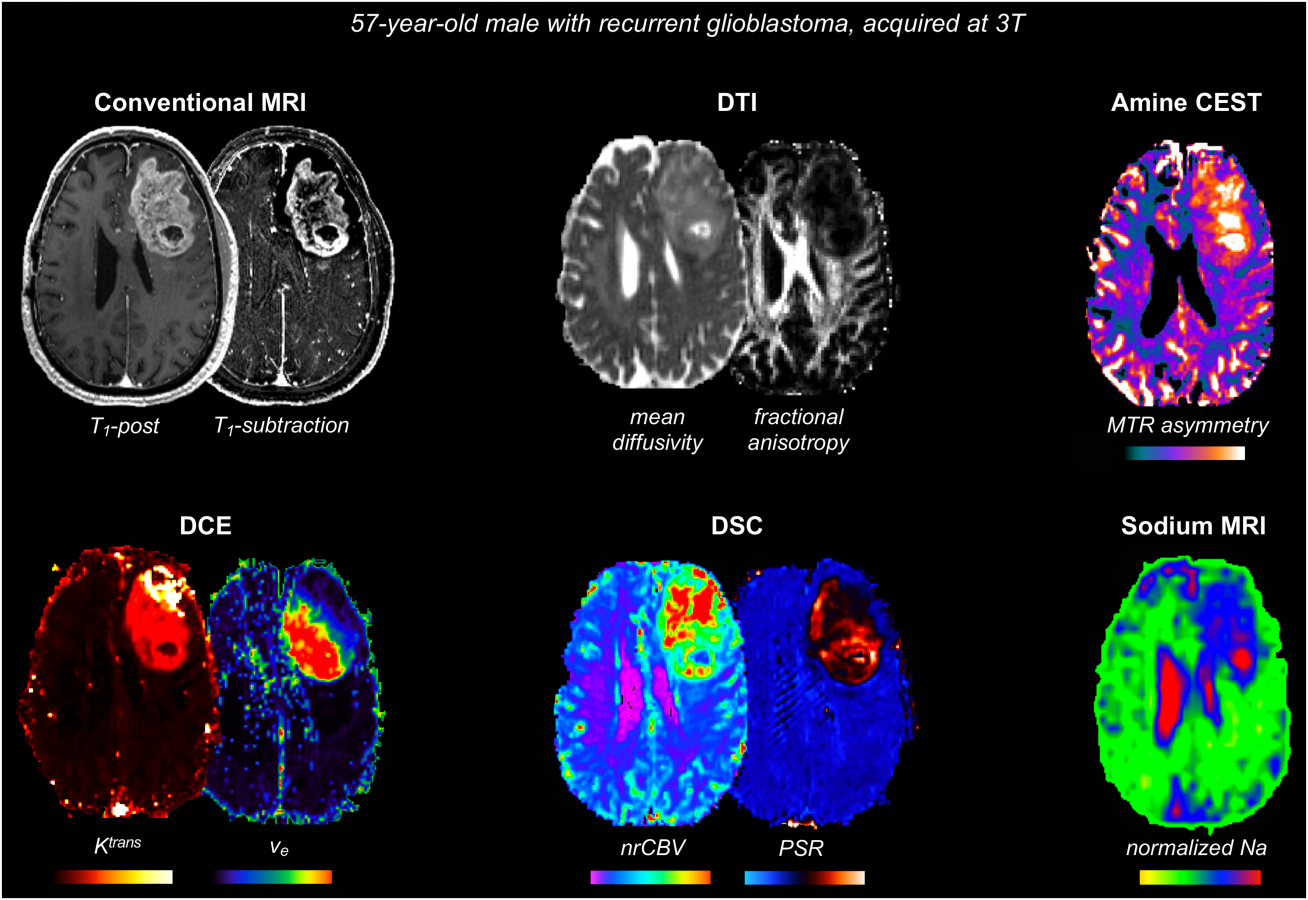
Representative case with additional advanced imaging acquisitions that can be integrated with BTIPs. DTI allows to compute fractional anisotropy and diffusivity, as well as perform tractography, if needed. Amine CEST can be employed to generate MTR asymmetry, reflecting tumor acidity. DSC maps are useful to assess tumor vasculature and angiogenesis (nrCBV) and tissue cytoarchitecture (PSR). DCE maps reflect the contrast leakage characteristics. In this case, DCE metrics were obtained simultaneously with DSC, using a dynamic spin-and-gradient-echo perfusion sequence as described in (81). Finally, sodium images were obtained at the end of the protocol and sodium signal voxel-wise was normalized to the eye sodium signal.

Figure 3 shows some examples of additional advanced sequences that can be integrated with BTIPs.

### 4.1. fMRI and DTI

fMRI and DTI (diffusion tensor imaging) may be acquired either for research projects or for presurgical planning (88), especially in gliomas. fMRI and DTI could be acquired as part of research protocols, for instance in studies investigating the value of DTI metrics for predicting treatment response (89) or in studies employing fMRI to evaluate neural plasticity and functional remapping (90). As for presurgical mapping, during the acquisition of BTIPs, the investigators usually do not have to include presurgical imaging, since clinical trials typically focus on systemic treatments initiated *after* surgery. In fact, radiologic evaluation of treatment response starts on post-surgical scans according to RANO (14) and on post-radiation scans according to mRANO (15) and RANO 2.0 (18). However, it may be useful to integrate presurgical fMRI and DTI during clinical trials when employing neoadjuvant treatments (in which patients can be imaged before surgery) or in case patients enrolled in clinical trials are considered for repeat surgery during follow-up. In both cases, it is possible that fMRI and DTI need to be integrated in BTIPs protocols for presurgical mapping purposes.

fMRI, either task-based or resting-state, should be acquired before contrast injection, since fMRI is based on T_2_*-weighted GRE-EPI sequences (91), similarly to DSC, and is therefore sensitive to T_1_ and T_2_* signal changes induced by gadolinium. fMRI acquisitions, tasks, and processing methods vary across institutions. The ASFNR guidelines propose to acquire fMRI with 3.4 × 3.4 mm^2^ in-plane resolution and 4–5 mm slice thickness, with six 10-volume blocks for tasks and six 10-volume blocks for rest, for a total of 120 EPI volumes.

DTI should be preferably acquired before contrast injection as well, in compliance to what is suggested for DWI in BTIPs. For completeness, it should be noted that there is evidence of contrast agent influencing DTI-metrics such as fractional anisotropy (92), while ADC measurements do not seem to be influenced by gadolinium (93, 94). The ASFNR guidelines for clinical DTI recommend to acquire DTI with a single-shot SE-EPI sequence, with isotropic voxels 2 × 2 × 2 mm^3^ at 3 T (2.5 × 2.5 × 2.5 mm^3^ at 1.5 T), and with three *b *= 0 images and at least 25 directions at *b *= 1,000 s/mm^2^ (https://www.asfnr.org/clinical-standards). Multicenter trials focusing on non-neoplastic diseases adopted similar parameters for DTI, with single-shell *b *= 1,000 s/mm^2^, 64 directions, and 2 × 2 × 2 mm^3^ isotropic voxels (95, 96). Other multicenter trials adopted similar protocols with some protocol variations, such as *b *= 700 s/mm^2^ (97) or b = 1,300 s/mm^2^ and voxel size 2.7 × 2.7 × 2.7 mm^3^ (98). Compared to BTIP-compliant DWI, these DTI sequences lack the *b *= 500 s/mm^2^ shell and have thinner slices, as well as a higher number of directions for diffusion-encoding gradients. The lack of *b *= 500 s/mm^2^ images may slightly affect the accuracy of ADC computation, while the thinner slices may decrease signal-to-noise ratio. However, in the case that DTI is integrated in BTIPs, it is overall reasonable to *replace* BTIP-compliant DWI with DTI (*b *= 1,000 s/mm^2^), as long as diffusion acquisitions are kept consistent in all follow-up scans of a given patient.

### 4.2. DCE

DCE (“permeability”) perfusion imaging is a dynamic T_1_-weighted imaging technique that is used in a variety of solid tumors to provide insight into vascular permeability. Although *not* included in BTIPs, DCE has applications in neuro-oncologic imaging. In the brain, DCE is thought to reflect alterations in the blood-brain barrier integrity (99). DCE-derived metrics such as K^trans^ have been employed for differential diagnosis (100), to predict and/or monitor treatment response (101–103), and to distinguish between treatment effects and tumor recurrence (75, 104), similar to DSC perfusion measures of CBV. The QIBA alliance has provided consensus recommendations for brain DCE (https://qibawiki.rsna.org/index.php/Profiles) (105). According to QIBA, DCE should consist of a dynamic 3D T_1_w SPGR with TE/TR minimal/3–8 ms, FA 10–15° at 3 T (25–35° at 1.5 T), ≤10 s temporal resolution (ideally ≤5 s) with at least 5 dynamics acquired before the bolus injection, acquisition time of ≥5 min, FOV 220–240 mm, matrix 256 × 128–160, slice thickness ≤5 mm. Additionally, pre-injection T_1_ mapping with the variable FA approach is suggested. As an example, a recent multicenter study employed a 6-minute long DCE sequence with ∼5 s temporal resolution and 2.4 × 2.4 × 2.5 mm^3^ voxel size (106, 107).

While some research strategies have included *both* DCE and DSC perfusion to estimate vascular permeability and volume, respectively, DCE requires a separate injection of GBCA in addition to the one needed for DSC. BTIP-compliant protocols that include DCE *and* DSC can therefore be achieved with two alternative strategies (11). The first strategy (1 + 1 scheme) is to administer a full dose of GBCA while acquiring DCE and then acquire post-contrast T_1_-weighted images followed by administration of an additional full dose for DSC. An alternative strategy (½ + ½ scheme) would be to acquire DCE with half dose of contrast agent, then DSC with another half dose bolus, and lastly the post-contrast T_1_-weighted images. While the 1 + 1 scheme provides higher contrast-to-noise ratio and accuracy in perfusion/permeability metrics, double dosage raises concerns for gadolinium deposition in gray matter nuclei (even though little is known about potential clinical implications of such deposition) (108) and for toxicity in patients with chronic kidney disease (109), also considering that brain tumor patients are followed with many longitudinal scans. Both the ½ + ½ and the 1 + 1 schemes are BTIP-compliant, and the choice should be made depending on the institution experience/preference and on the type of GBCA employed, as for instance macrocyclic agents are possibly linked with a very low risk of nephrogenic systemic fibrosis. If the ½ + ½ scheme is preferred, a 30° FA should be set for DSC, which is more adherent to BTIP recommendations, while 60° FA should be preferred in case of full preload injected during DCE (1 + 1 scheme). Finally, it is worth mentioning that some studies proposed to employ dual-echo DSC to perform simultaneous DSC and DCE with a single bolus of GBCA and a single acquisition (87, 110), which is a potential future approach to obtain both DSC and DCE while limiting GBCA doses and reducing acquisition time.

### 4.3. ASL, spectroscopy, CEST, sodium imaging, and SWI

ASL (arterial spin labeling) is a technique that enables estimation of cerebral blood flow (CBF) by imaging the magnetically labeled inflowing blood (111), and it has been increasingly employed in brain tumor imaging studies as a perfusion technique (112–114). Similarly to DSC-derived CBV, ASL-derived CBF has also been shown to correlate with microvascular density (115). According to current guidelines (116, 117), ASL should be performed with either pseudo-continuous or pulsed labeling, with a post-labeling delay (or inversion time) of 2,000 ms in the clinical adult population, in-plane resolution of 3–4 mm, and slice thickness of 4–8 mm. As discussed in a recent article, exogenous GBCA administration causes significant signal loss in ASL, as GBCA-induced T_1_ shortening results in a rapid decay in the magnetic labeling of blood spins (118). Therefore, ASL sequences should be integrated in BTIPs before contrast injection.

MR spectroscopy (MRS) techniques are traditionally employed in brain tumor MRI mainly to assess the tissue concentration of creatine, choline, and n-acetylaspartate. More recently, MRS techniques to detect 2HG in gliomas have been optimized in some institutions (119–121). 2HG is a product of mutant IDH, and its detection is not only useful for IDH profiling at diagnosis, but also for the longitudinal evaluation of IDH inhibition (122). MRS sequences may be integrated with BTIPs in LGG clinical trials testing IDH inhibitors, in order to correlate clinical outcomes with longitudinal changes in 2HG. For non-enhancing lesions—e.g., most of IDH-mutant gliomas—MRS can be acquired either before or after GBCA administration, since no significant amount of GBCA is thought to be present in the extravascular extracellular space in such cases. For enhancing lesions, it is debatable whether MRS should be integrated in BTIPs before or after GBCA. In single-voxel MRS, the administration of GBCA allows to more accurately position the voxel on the enhancing component of the lesion, and better avoid sampling cystic or necrotic areas or vasogenic edema. However, distortions in the local magnetic field induced by GBCA susceptibility may potentially affect the accuracy of MRS measurements, particularly in long-TE protocols. Evidence in this regard is inconclusive, as some data advocate for GBCA *not* affecting the quantification of creatine, choline, and n-acetylaspartate (123), while other findings showed a choline underestimation only when using negatively-charged types of GBCA (e.g., gadoteric acid) (124). Overall, common sense suggests that partial volume effects due to necrotic/cystic/edematous tissue sampling may impact on measurement accuracy more than GBCA effects. Therefore, it is overall reasonable to acquire single-voxel MRS after GBCA administration—as often happens in the clinical practice –, as long as consistency is maintained across subjects and timepoints, and the GBCA type is reported in the article. Conversely, multi-voxel MRS is less impacted by an accurate voxel positioning, and is perhaps more feasible before GBCA.

Amine-weighted CEST (chemical exchange saturation transfer) and APT (amide proton transfer) CEST are techniques that have been proposed to sample tumor acidity (with a contribution to the signal dependent on protein content, especially in case of APT), providing insights into tumor metabolism, with potential applications in monitoring treatment response (125–127). CEST MRI is typically performed at 3 T field strength or higher, and should be integrated in BTIPs before contrast injection for consistency with the common practice.

Sodium MRI has been recently included in imaging protocols in some institutions, as it is a promising technique to study tumor electrolyte homeostasis, with potential insights into tissue biology and susceptibility to treatments (128–130). Sodium imaging can be integrated in BTIPs either before or after contrast injection, since there is evidence that the estimation of total sodium concentration should not be impacted by gadolinium (131).

SWI (susceptibility-weighted imaging) may be helpful in brain tumor imaging at diagnosis. Identifying intra-tumoral susceptibility signals may be helpful for differential diagnosis since they can be present in gliomas due to microhemorrhage or microcalcification, while they are substantially less frequent in PCNSL. Additionally, the evaluation of phase images can differentiate microcalcifications from microhemorrhage, which may aid the molecular prediction of oligodendrogliomas (132). Overall, the role of SWI in treatment response assessment seems marginal at the moment. For the aforementioned applications, SWI should be integrated in BTIPs before contrast agent administration. Post-contrast SWI, on the other hand, may have other applications, such as the evaluation of vascular malformations (133).

## 5. Future directions and conclusions

BTIPs overall lean towards conventional imaging (T_1_, T_2_, and FLAIR) acquisitions being performed with 3D imaging, with the caveat that 3D imaging can be more technically challenging and should be used when yields sufficient image quality. As discussed, 3D images are useful for adjusting the tilt of image planes, for multiplanar reconstructions, and for volumetric assessments of lesion volumes. Whether lesion size should be assessed through linear measurements (unidimensional 1D, or bidimensional 2D) or volumetric segmentations (3D) in clinical trials has been object of debate. Currently, both 2D and 3D assessments are contemplated in RANO 2.0 (18). Some studies showed good agreement between linear and volumetric measurements, and advocated for 2D being non-inferior to 3D when predicting overall survival (134–137). However, as already mentioned, evidence shows that measuring lesion size through 3D segmentations can reduce the inter-observer variability compared to 1D and 2D evaluations (138–144), and have smaller bias and variability for the measurement of nodules (145). As for the impact on response assessment, some studies in slower growing tumor show significant differences in progression-free survival between volumetric and 1D/2D methods (41, 134), and studies on malignant gliomas reported that only volumetric measures of tumor size were predictive of survival (146). Finally, 3D segmentations aid the quantification of tumor size in peculiar scenarios where the disease is considered non-measurable according to RANO-compliant 1D/2D measurements, for instance in the case of rim enhancement surrounding a cyst or surgical cavity. Overall, the current body of literature suggests that 3D measurements for tumor burden quantification is *equal or better* than 2D measurements. Therefore, adopting 3D measurements for the quantification of tumor burden is advisable, if technically feasible. Currently, the main technical factors limiting the application of volumetric assessments are that tumor segmentations are time-consuming and require dedicated software (often not available on PACS systems). A potential resource to overcome these obstacles and eventually make volumetric assessments more widely feasible will be the use of artificial intelligence (AI) for lesion segmentation, which has the potential to yield automated tumor masks without the need of human intervention (147). One recent study showed that PFS computed from AI segmentations on clinical trials datasets was comparable to the human centralized review read, while the local human read yielded different PFS, possibly due to training and expertise differences in the local institutions (148). Similarly, another study showed how an automatic assessment of RANO (AutoRANO) is feasible through AI (149). As of today, both open access and commercial AI segmentation tools are beginning to become more and more available, with variable performance. A general trend towards 3D acquisitions will contribute to speeding up the process of obtaining more longitudinal brain tumor imaging datasets to train AI, thereby improving their performance, and consequently paving the way for a future use of AI segmentations in clinical trials.

Another aspect that has been encouraged in BTIP articles is the generation of T_1_-subtraction maps. T_1_-subtraction maps are overall a more accurate approach to identify the enhancing tissue compared to a separate side-by-side qualitative evaluation of T_1_-pre and T_1_-post, and are essential in some otherwise difficult cases. One such case is the identification of subtle degree of enhancement, for instance in tumors treated with anti-angiogenic therapy, for which the use of T_1_-subtraction maps has been shown to improve the quantification of tumor burden and the predictive value of the radiologic assessment with regards to overall survival (25). Additionally, T_1_-subtraction maps are helpful to sort out inherent T_1_-pre hyperintensity due to hemorrhage (in glioblastoma, mostly), melanin (in melanoma BM), or calcification. In this regard, the voxel-wise subtraction is not only able to exclude from the enhancing voxels the ones with inherent T_1_-hyperintensity but also to capture potential enhancement in areas of inherent T_1_-hyperintensity, where T_1_ signal is high before contrast and even higher after contrast. As of today, T_1_-subtraction maps are obtainable quite easily in neuro-imaging laboratories, and could be generated during the central review of the scans in clinical trials. Conversely, they are more challenging to generate in clinical settings, since the normalization and co-registration of T_1_-pre and T_1_-post warranted before voxel-wise subtraction are commonly not feasible on the scanner nor on PACS at this time. A wider use of T_1_-subtraction maps and further evidence of their advantages may heighten interest towards the development of integrated software tools for their clinical implementation.

As for the efforts towards a better standardization of pulse sequence parameters across institutions, a remarkable advancement may be represented by synthetic MRI (SyMRI), using a technique named quantification of relaxation times and proton density by multi-echo acquisition of a saturation-recovery using turbo spin-echo readout (QRAPMASTER) (150). This technique estimates T_2_, T_1_, and PD values voxel-wise by fitting the Bloch equations to a QRAPMASTER SyMRI sequence: T_2_ values are computed from the multiple echoes acquired, T_1_ values from the saturation pulses acquired with different delays, and PD values by extrapolating the signal intensity at TE zero (151). This approach not only allows to obtain quantitative voxel-wise maps of T_1_, T_2_, and PD, but also to generate T_1_-, T_2_-weighted and T_2_-weighted FLAIR images with arbitrary TE, TR, and TI. This would potentially enable to more easily generate images with uniform pulse sequence parameters from multi-centric acquisitions. SyMRI is already available on some commercial scanners (e.g., “SyntAc” on Siemens, “MAGiC” on GE), and some studies advocate for the accuracy of SyMRI in parameter quantification (152) and for its potential usefulness in gliomas (151). An alternative approach to improve the comparability of images across institutions and manufacturers may be represented by standardization methods applied during post-processing (153), possibly with the aid of AI (154).

Finally, a number of novel approaches have been proposed to accelerate image acquisition with an enhanced exploitation of parallel imaging (155), including parallel imaging with multi-slice techniques (156) and controlled aliasing techniques (157), as well as deep learning reconstruction (158, 159). A promising example in this regard is the wave-CAIPI acceleration method, which has been shown to generate images with quality comparable to conventional acceleration methods in approximately one third of the time (acquisition times: 1min14s for 3D IR-GRE T_1_, 1min19s for 3D T_2_-weighted TSE, 2 min for 3D T_2_-weighted FLAIR, 1min29s for SWI) (160). Advancements in the field of accelerated image acquisition have the potential to reduce the acquisition time for BTIPs, which would alleviate the burden for the patient and improve flexibility in scan planning. Additionally, faster sequences also have the potential of reducing motion artifacts, especially in oncologic patients who cannot always be fully compliant.

In conclusion, imaging protocols complying with BTIPs should be implemented for clinical trials, with some degree of flexibility to accommodate institutional preferences that do not conflict with treatment response assessments (e.g., DTI in place of DWI). For the clinical routine, imaging centers can use BTIPs as suggestions for imaging protocols in brain tumors, keeping in mind that most BTIP recommendations are conceived for standardization and feasibility in multicenter clinical trials, and therefore the single institutions may need to adapt them to their clinical needs. More in general, obtaining standardized imaging datasets in brain tumor patients is not only a need for reliably assessing treatment response in clinical trials, but also a precious resource for future imaging studies which would retrospectively analyze such datasets. When implementing imaging protocols in neuro-oncology, general concepts regarding dosage and timing of GBCA administration, as well as optimal pulse sequence parameters, should be taken into consideration.
